# Supplementary material for: Using Genomic Variation to Distinguish Ovarian High-Grade Serous Carcinoma from Benign Fallopian Tubes
Source: Int J Mol Sci. 2022 Nov 26;23(23):14814. doi: 10.3390/ijms232314814 (PMC9738935; doi:10.3390/ijms232314814)
Supplement: Supplementary file 1 [file ijms-23-14814-s001.zip › ijms-1998691-supplementary.pdf]

International Journal of
*Molecular Sciences*

*Article*

# Using Genomic Variation to Distinguish Ovarian High-Grade Serous Carcinoma from Benign Fallopian Tubes

**Jesus Gonzalez-Bosquet [1,*], Nicholas D. Cardillo [2], Henry D. Reyes [3], Brian J. Smith [4], Kimberly K. Leslie [5], David P. Bender [1], Michael J. Goodheart [1] and Eric J. Devor [1]**

1 Department of Obstetrics and Gynecology, University of Iowa, 200 Hawkins Dr., Iowa City, IA 52242, USA
2 Hanjani Institute of Gynecologic Oncology, Thomas Jefferson University, Philadelphia, PA 19107, USA
3 Department of Obstetrics and Gynecology, University of Buffalo, Buffalo, NY 14203, USA
4 Department of Biostatistics, University of Iowa, 145 N Riverside Dr., Iowa City, IA 52242, USA
5 Division of Molecular Medicine, Departments of Internal Medicine and Obstetrics and Gynecology, The University of New Mexico Comprehensive Cancer Center, 915 Camino de Salud, CRF 117, Albuquerque, NM 87131, USA
* Correspondence: jesus-gonzalezbosquet@uiowa.edu; Tel.: +1-(319)-356-2160; Fax: +1-(319)-353-8363

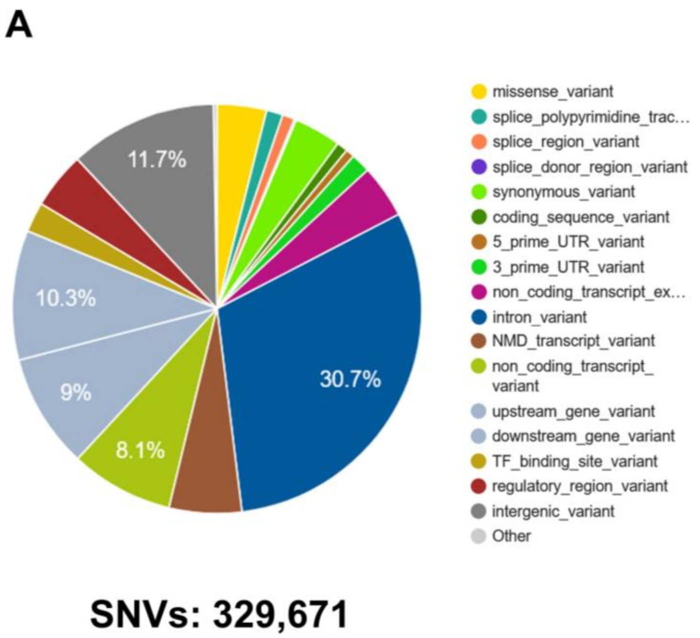


**B**

| Consequence type | Count |
| --- | --- |
| splice_acceptor_variant | 184 |
| splice_donor_variant | 197 |
| stop_gained | 266 |
| splice_donor_5th_base_variant | 179 |
| frameshift_variant | 188 |
| stop_lost | 49 |
| start_lost | 48 |
| inframe_insertion | 37 |
| inframe_deletion | 253 |
| missense_variant | 20,360 |
| protein_altering_variant | 2 |
| splice_polypyrimidine_tract_variant | 4,242 |
| splice_region_variant | 1,283 |
| incomplete_terminal_codon_variant | 2 |
| splice_donor_region_variant | 585 |
| synonymous_variant | 17,083 |
| stop_retained_variant | 16 |
| coding_sequence_variant | 8,324 |
| mature_miRNA_variant | 6 |
| 5_prime_UTR_variant | 3,424 |
| 3_prime_UTR_variant | 4,439 |
| non_coding_transcript_exon_variant | 20,458 |
| intron_variant | 63,304 |
| non_coding_transcript_variant | 1 |
| upstream_gene_variant | 9,128 |
| downstream_gene_variant | 3,663 |
| TF_binding_site_variant | 6,641 |
| regulatory_region_variant | 17,139 |
| intergenic_variant | 148,170 |

**Supplementary Figure S1.** Analysis of all SNVs from one of the HGSC samples analyzed with VEP (Variant Effect Predictor) which predicts the functional effects of genomic variants. **A.** Distribution of all predicted functional consequences of the SNVs found in pie format. **B.** Same variants in table format to report all SNVs numbers (some SNVs may be in several categories).

**A**

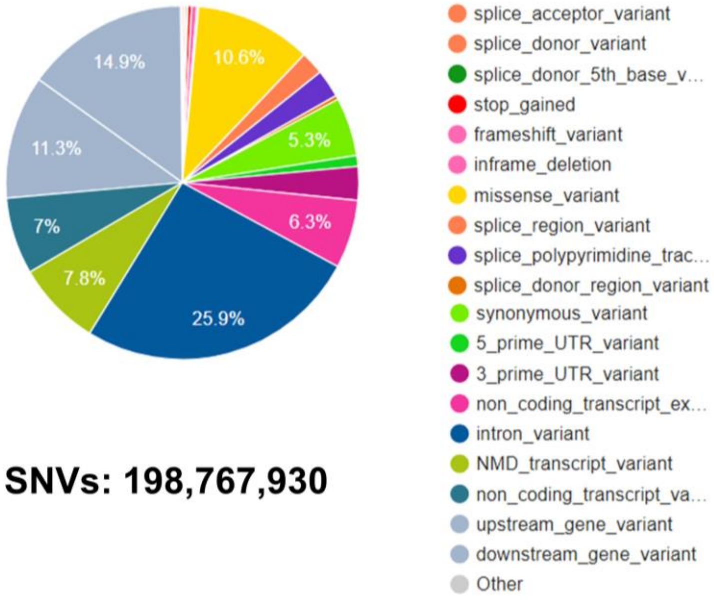


**B**

| Consequence Type | Count |
|---|---|
| transcript_ablation | 23 |
| splice_acceptor_variant | 349,294 |
| splice_donor_variant | 374,368 |
| splice_donor_5th_base_variant | 298,585 |
| stop_gained | 650,535 |
| frameshift_variant | 970,774 |
| stop_lost | 30,535 |
| start_lost | 69,805 |
| inframe_insertion | 133,479 |
| inframe_deletion | 323,810 |
| protein_altering_variant | 10,506 |
| missense_variant | 21,127,593 |
| splice_region_variant | 4,234,450 |
| splice_polypyrimidine_tract_variant | 5,112,132 |
| splice_donor_region_variant | 731,012 |
| incomplete_terminal_codon_variant | 5,383 |
| synonymous_variant | 10,632,989 |
| stop_retained_variant | 14,845 |
| start_retained_variant | 1,033 |
| coding_sequence_variant | 43,894 |
| mature_miRNA_variant | 12,216 |
| 5_prime_UTR_variant | 2,000,244 |
| 3_prime_UTR_variant | 6,099,521 |
| non_coding_transcript_exon_variant | 12,445,778 |
| intron_variant | 51,485,930 |
| NMD_transcript_variant | 15,468,345 |
| non_coding_transcript_variant | 13,963,897 |
| upstream_gene_variant | 22,481,881 |
| downstream_gene_variant | 29,678,694 |
| intergenic_variant | 16,379 |

**Supplementary Figure S2.** Analysis of all GNOMAD WES population SNVs with VEP. **A.** Distribution of all predicted functional consequences of SNVs in pie format. **B.** Same data in table format to report all SNVs numbers by their downstream effects.

**Supplementary Table S1**: Pathway analysis of genes including 16,636 variants. Pathway enrichment analysis were performed with *clusterProfiler*, R package, which interrogates *KEGG* database. Represented are significant pathways with their ID, description, p-value and adjusted p-value for multiple comparisons.

| ID | Description | p.adjust | geneID |
|---|---|---|---|
| hsa04974 | Protein digestion and absorption | <0.001 | CELA3A/COL11A1/COL4A3/COL6A6/SLC36A1/ATP1A4/SLC3A1/COL4A4/COL25A1/COL23A1/COL11A2/COL12A1/PRSS2/COL5A1/COL17A1/COL4A1/COL4A2/COL6A1/CELA3B/COL9A2/COL24A1/SLC8A1/COL5A2/COL6A3/SLC36A2/COL21A1/SLC16A10/COL28A1/COL26A1/COL14A1/COL22A1/COL27A1/ATP1B2/COL1A1/COL6A2/COL4A6/PRSS1/PRCP/CPB2/COL5A3/COL20A1/COL18A1/ATP1A2/SLC9A3/SLC6A19/SLC36A3/MEP1A/COL19A1/COL13A1/CTRB1/KCNN4/COL2A1/SLC7A7/SLC15A1/ATP1A3/COL9A1/ACE2/CELA2A/SLC1A5 |
| hsa04070 | Phosphatidylinositol signaling system | <0.001 | PIK3C2B/INPP4B/DGKB/PI4KA/ITPR1/CDS1/SYNJ2/MTMR7/PLCE1/MTMR6/PLCB2/PLCG2/DGKE/PIK3R3/CALM2/PIKFYVE/PIK3R1/DGKI/PIP5K1B/PIK3C2A/PIK3C2G/PIP4K2C/DGKH/SYNJ1/MTM1/MTMR1/PLCD1/PIK3CB/DGKG/CALML4/PRKCA/PI4K2B/MTMR2/INPP5B/PIP5K1A/INPP4A/DGKD/ITPR3/IP6K3/ITPR2/ITPK1/ITPKA/PRKCB/PIK3C3/PIP5K1C/CDS2/MTMR3/CALML5/CALM1/PIK3R2/IMPA2/IP6K1/CALM3/PLCB1/IP6K2/PLCZ1 |
| hsa04512 | ECM-receptor interaction | <0.001 | COL4A3/COL6A6/FRAS1/LAMA5/ITGB6/ITGAV/FN1/COL4A4/DMP1/SV2C/LAMA2/THBS2/FREM1/COL4A1/COL4A2/ITGA11/LAMA1/LAMA3/COL6A1/COL9A2/TNR/COL6A3/ITGA2/THBS4/CD36/RELN/LAMC3/VWF/COL1A1/COL6A2/COL4A6/HSPG2/LAMC1/LAMA4/LAMB1/ITGB1/AGRN/TNN/LAMB3/SDC1/ITGB5/ITGA1/ITGB8/ITGA8/ITGB3/ITGB4/SDC4/NPNT/COL2A1/ITGA2B/COL9A1 |
| hsa04912 | GnRH signaling pathway | <0.001 | PRKACB/PLA2G4A/CACNA1C/SOS2/GRB2/ITPR1/MAPK10/ADCY2/CAMK2B/PTK2B/ADCY4/PLCB2/ADCY9/CALM2/CACNA1D/MAP3K1/MAPK9/PLA2G4D/PLA2G4F/GNAS/CACNA1S/SOS1/ADCY5/ADCY1/CAMK2G/CALML4/PRKCA/NRAS/MAP3K2/RAF1/CAMK2A/ITPR3/MAPK14/ITPR2/PLA2G4E/PRKCB/MMP2/CALML5/KRAS/MAP3K4/MAPK12/PRKCD/CALM1/FSHB/MAPK1/GNA11/GNAQ/CALM3/PLCB1/ADCY8/PLD2/PLA2G4C |
| hsa05412 | Arrhythmogenic right ventricular cardiomyopathy | <0.001 | CACNA1C/RYR2/ITGB6/ITGAV/LAMA2/CACNA2D1/CACNB2/PKP2/ITGA11/LAMA1/ACTN2/SLC8A1/TCF7L1/CACNA1D/CACNA2D3/ITGA2/CTNNA3/CDH2/DSC2/DMD/CACNA1S/LEF1/CTNNA1/DSP/ITGB1/SGCA/CACNG6/CACNG1/CTNNA2/ITGB5/ITGA1/ITGB8/ITGA8/CACNA2D4/JUP/ITGB3/ITGB4/DSG2/ATP2A3/ATP2A1/ITGA2B/CACNG2/CACNB4/DES |
| hsa04925 | Aldosterone synthesis and secretion | <0.001 | PRKACB/CACNA1C/ATP1A4/ATP2B4/ITPR1/ADCY2/CAMK2B/ADCY4/PRKD1/PLCB2/CACNA1H/ADCY9/CACNA1G/CACNA1I/CALM2/CACNA1D/ATP1B2/GNAS/CACNA1S/PRKD3/PRKCE/ADCY5/CAMK4/CREB5/ADCY1/CAMK1D/CAMK2G/CALML4/PRKCA/ATP1A2/ATP2B2/CAMK2A/ITPR3/DAGLB/CREB3L2/CREB3L1/ITPR2/PRKCB/CALML5/DAGLA/CALM1/CREB3L3/ATP2B1/ATP1A3/CYP11B2/CYP11A1/GNA11/GNAQ/LDLR/CALM3/PRKD2/PLCB1/ADCY8 |
| hsa04510 | Focal adhesion | <0.001 | COL4A3/MYLK/COL6A6/RAP1B/SOS2/GRB2/SHC2/PAK4/LAMA5/VAV3/ROCK2/ITGB6/ITGAV/FN1/COL4A4/MAPK10/FLT4/LAMA2/THBS2/RAC1/TLN1/VAV2/COL4A1/COL4A2/ITGA11/LAMA1/LAMA3/COL6A1/COL9A2/PIK3R3/TNR/COL6A3/FLNB/PDGFRA/KDR/ITGA2/PIK3R1/THBS4/MAPK9/RELN/PIP5K1B/LAMC3/DOCK1/PARVA/VWF/PAK6/COL1A1/MYL12A/ACTN4/COL6A2/COL4A6/LAMC1/PPP1R12B/SOS1/PIK3CB/PAK2/LAMA4/LAMB1/CAV1/ITGB1/PDGFD/PRKCA/CRKL/PIP5K1A/TNN/LAMB3/AKT3/RAF1/ITGB5/ITGA1/MYLK4/ITGB8/ITGA8/PAK1/PRKCB/ITGB3/ITGB4/PIP5K1C/FLT1/RASGRF1/ARHGAP35/CCND3/COL2A1/ITGA2B/VAV1/PIK3R2/MYLK3/ERBB2/MAPK1/CRK/RHOA/COL9A1/FYN/VASP |
| hsa04750 | Inflammatory mediator regulation of TRP channels | 0.001 | PRKACB/PLA2G4A/ALOX12/ITPR1/IL1RAP/MAPK10/ADCY2/CAMK2B/TRPA1/ADCY4/PLCB2/ADCY9/PLCG2/TRPV3/PIK3R3/CALM2/IL1B/PIK3R1/MAPK9/PRKCH/PLA2G4D/PLA2G4F/TRPV1/GNAS/NTRK1/PRKCE/ADCY5/PIK3CB/ADCY1/CAMK2G/CALML4/PRKCA/PLA2G6/KNG1/CAMK2A/ITPR3/MAPK14/ITPR2/PLA2G4E/PRKCB/CALML5/MAPK12/PRKCD/CALM1/PIK3R2/ASIC1/GNAQ/CALM3/PLCB1/ADCY8/BDKRB2/PLA2G4C |
| hsa04921 | Oxytocin signaling pathway | 0.001 | PRKACB/PLA2G4A/MYLK/CACNA1C/NFATC1/PRKAA2/RYR2/ROCK2/ITPR1/ADCY2/MEF2C/CAMK2B/CACNA2D1/CACNB2/ADCY4/RYR3/PLCB2/ADCY9/CALM2/CACNA1D/CACNA2D3/GUCY1A1/PRKAA1/NFATC4/PLA2G4D/PLA2G4F/MAP2K5/PIK3R5/RYR1/GNAS/TRPM2/CACNA1S/PPP1R12B/ADCY5/CAMK4/ADCY1/GNAI1/PRKAG2/CAMK1D/CAMK2G/CALML4/PRKCA/CACNG6/CACNG1/NRAS/RAF1/CAMK2A/MYLK4/ITPR3/PIK3CG/CACNA2D4/ITPR2/CAMKK2/PLA2G4E/PRKCB/GNAO1/NFATC2/CDKN1A/CALML5/KRAS/KCNJ18/CACNG2/CACNB4/CALM1/MYLK3/MAPK1/RHOA/GNAQ/PIK3R6/CALM3/PLCB1/OXTR/ADCY8/PLA2G4C |
| hsa04072 | Phospholipase D signaling pathway | 0.001 | PLA2G4A/DGKB/SOS2/TSC2/GRB2/SHC2/ADCY2/GRM1/PTK2B/ADCY4/ARF6/PLCB2/ADCY9/PLCG2/DGKE/CYTH1/INSR/LPAR4/MTOR/PIK3R3/GRM7/PDGFRA/GAB1/PIK3R1/GNA12/DGKI/AGPAT5/PIP5K1B/F2/AVPR1A/DGKH/PLA2G4D/PLA2G4F/PIK3R5/GNAS/SOS1/ADCY5/PIK3CB/DGKG/ADCY1/PDGFD/PRKCA/AVP/RHEB/GRM8/NRAS/PIP5K1A/AKT3/RALB/DGKD/RAF1/PIK3CG/RALGDS/PLA2G4E/PIP5K1C/KRAS/TSC1/PIK3R2/GRM4/MAPK1/FCER1A/RAPGEF4/RHOA/FYN/PIK3R6/PLPP2/PLCB1/ADCY8/INS/PLD2/PLA2G4C |

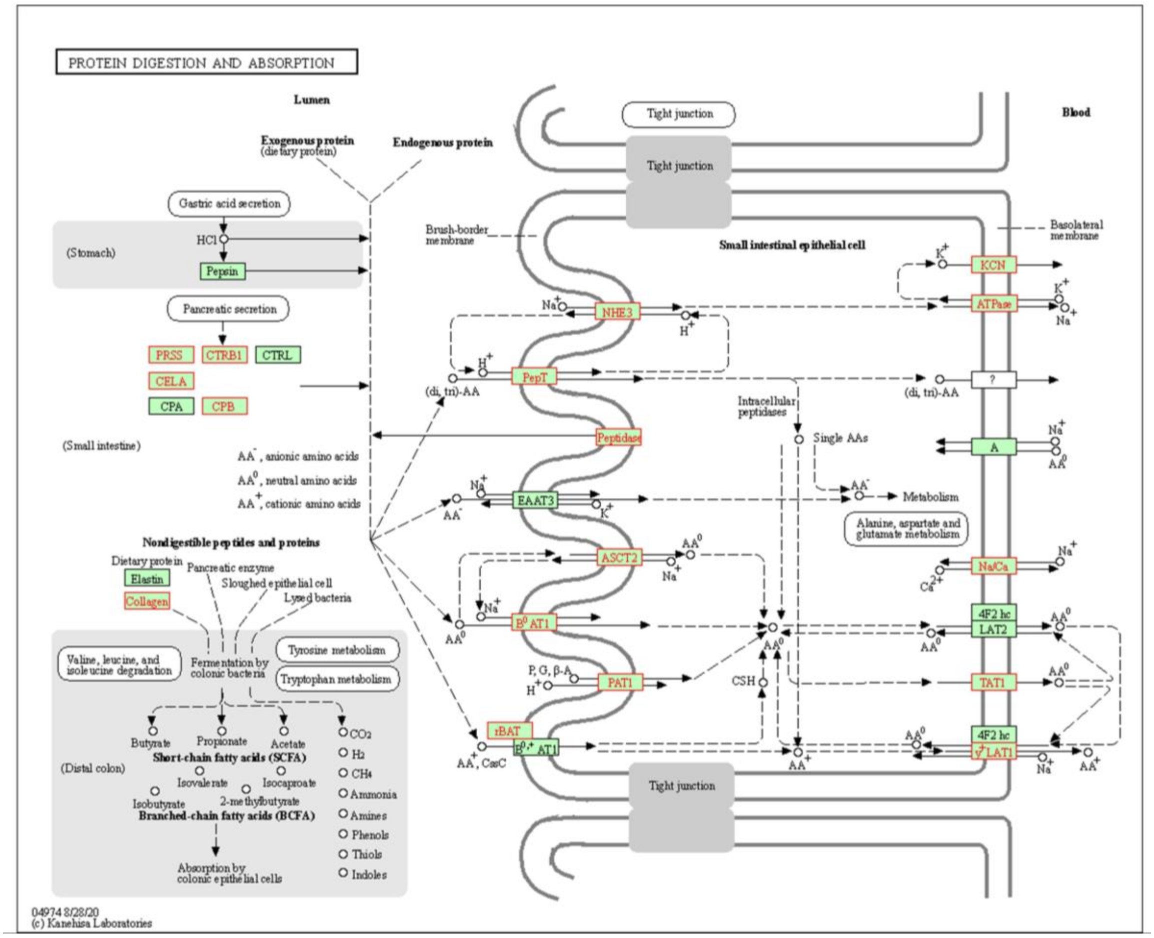


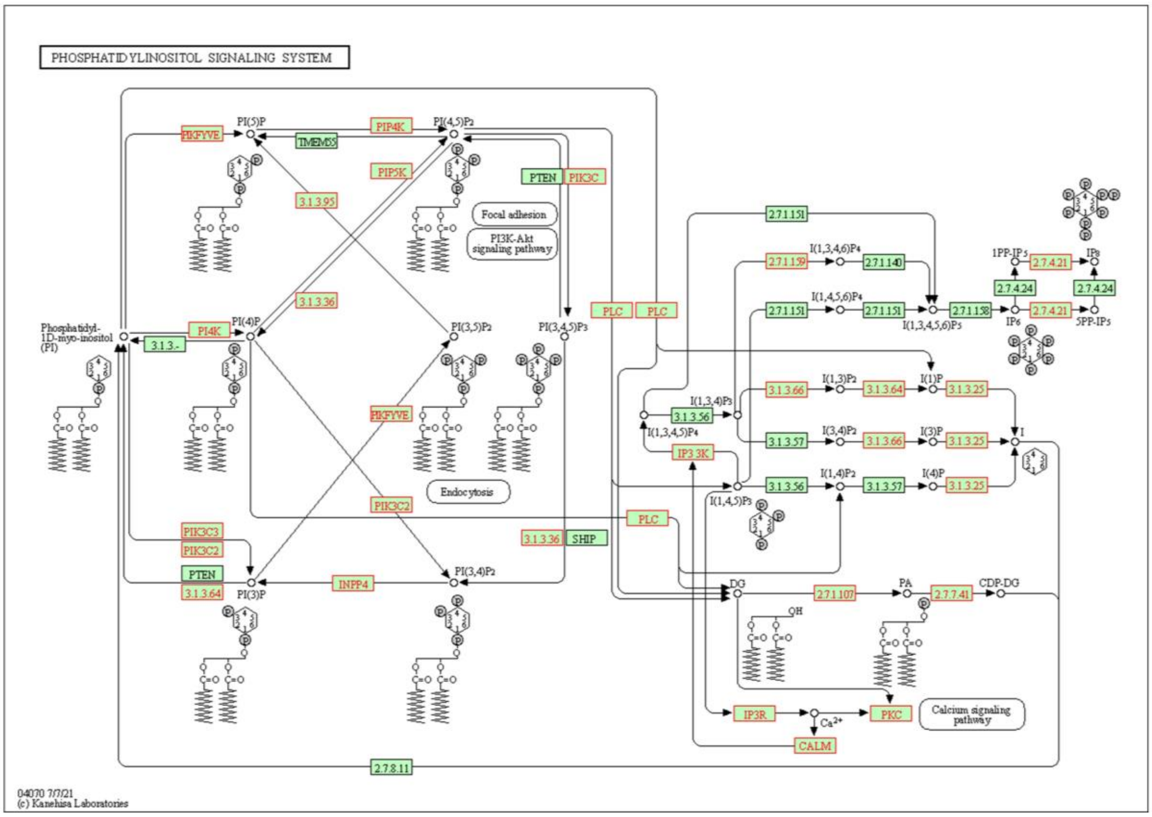


**Supplementary Figure S3.** Significant pathways 'Protein digestion and absorption' and 'Phosphatidylinositol signaling system' from KEGG database (adjusted p-value for multiple comparisons: <0.001)(printed with copyright permission of KEGG).

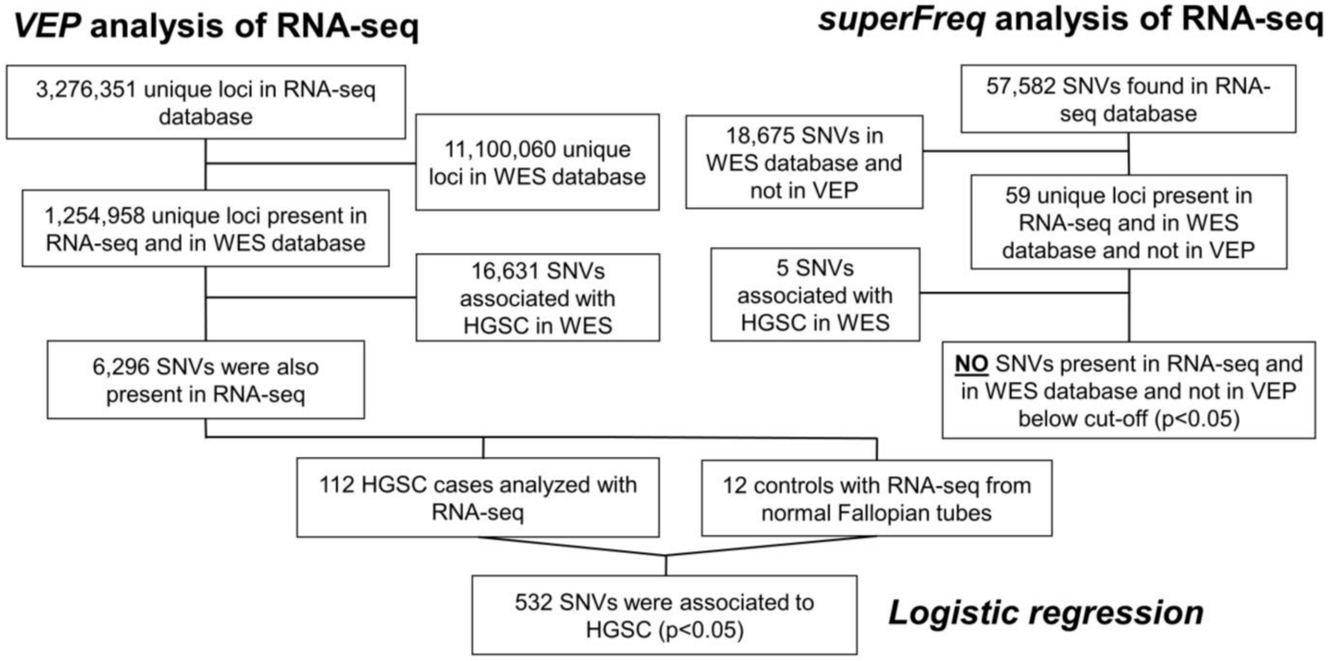


**Supplementary Figure S4.** Validation analysis in RNA-seq data**.**

**Variation analysis with VEP in RNA-seq data.** Of the initial more than 3 million SNVs found in all HGSC samples, over 1.2 million were also present in WES VEP analysis. Out of the initial 16,631 selected SNVs, associated with HGSC in the WES VEP analysis, 6,296 SNVs were also present in RNA-seq VEP analysis (Fig. 1). Unrelated controls consisted in 12 RNA-seq samples from the distal part of the Fallopian tube (fimbria) from patients with no disease and no family history of ovarian cancer. It resulted in 532 SNVs associated with HGSC (p-value < 0.05).

**Variation analysis with superFreq in RNA-seq data**. Of the initial more than 57 thousand SNVs found in all HGSC samples after quality filters, 59 were also present in WES superFreq analysis (Fig. 1). None of these SNVs passed the cut-off after the initial univariate logistic analysis.

**Supplementary Table S2**: Pathway analysis of genes including 532 variants. Pathway enrichment analysis were performed with *clusterProfiler*, R package, which interrogates *KEGG* database. Represented are significant pathways with their ID, description, p-value and adjusted p-value for multiple comparisons.

| ID | Description | pvalue | p.adjust | Symbol |
|---|---|---|---|---|
| hsa04068 | FoxO signaling pathway | <0.001 | 0.013 | INSR/TGFB3/HOMER2/FBXO32/PIK3R1/MDM2/GRB2/RAF1/FOXO3/ATM/MAPK10/USP7 |
| hsa04912 | GnRH signaling pathway | <0.001 | 0.013 | CALM2/CALML4/GRB2/ADCY8/RAF1/MAP3K1/PLCB1/PLA2G4A/MAPK10/ADCY4 |
| hsa04218 | Cellular senescence | <0.001 | 0.013 | HLA-F/CALM2/TGFB3/CDK1/PIK3R1/MRE11/MDM2/CALML4/RAF1/FOXO3/NFATC2/ATM/HLA-A |
| hsa04072 | Phospholipase D signaling pathway | <0.001 | 0.023 | INSR/RALGDS/PIK3R1/FYN/GRB2/ADCY8/RAF1/PLCB1/CYTH1/PLPP2/PLA2G4A/ADCY4 |
| hsa04015 | Rap1 signaling pathway | 0.001 | 0.045 | INSR/CALM2/MAGI1/RALGDS/PIK3R1/CALML4/FARP2/ADCY8/RAF1/DOCK4/PLCB1/ANGPT2/FLT4/ADCY4 |
| hsa04660 | T cell receptor signaling pathway | 0.001 | 0.049 | PIK3R1/FYN/GRB2/RAF1/NFATC2/MAPK10/TEC/CD4/RASGRP1 |
| hsa05210 | Colorectal cancer | 0.001 | 0.049 | BAK1/RALGDS/TGFB3/PIK3R1/GRB2/RAF1/MAPK10/MSH3 |
| hsa05163 | Human cytomegalovirus infection | 0.002 | 0.049 | HLA-F/CALM2/BAK1/PIK3R1/MDM2/CALML4/GRB2/ADCY8/RAF1/NFATC2/PLCB1/CASP8/ADCY4/HLA-A |
| hsa05203 | Viral carcinogenesis | 0.002 | 0.049 | HLA-F/BAK1/PSMC1/CDK1/PIK3R1/MDM2/GSN/SND1/GRB2/CASP8/USP7/KAT2B/HLA-A |
| hsa05205 | Proteoglycans in cancer | 0.002 | 0.049 | ANK3/EIF4B/ESR1/IQGAP1/WNT5B/ERBB4/PIK3R1/MDM2/PPP1R12B/GRB2/RAF1/EZR/GPC1 |
| hsa01524 | Platinum drug resistance | 0.002 | 0.049 | BAK1/PIK3R1/MDM2/REV3L/ATM/CASP8/MSH3 |
| hsa04915 | Estrogen signaling pathway | 0.002 | 0.049 | CALM2/ESR1/PIK3R1/CALML4/GRB2/ADCY8/RAF1/FKBP5/PLCB1/ADCY4 |
| hsa05170 | Human immunodeficiency virus 1 infection | 0.003 | 0.049 | HLA-F/CALM2/BAK1/CDK1/PIK3R1/CALML4/RAF1/NFATC2/ATM/CASP8/MAPK10/CD4/HLA-A |
| hsa05214 | Glioma | 0.003 | 0.049 | CALM2/BAK1/PIK3R1/MDM2/CALML4/GRB2/RAF1 |
| hsa04935 | Growth hormone synthesis, secretion and action | 0.003 | 0.049 | PIK3R1/GRB2/ADCY8/RAF1/MAP3K1/PLCB1/GHR/MAPK10/ADCY4 |
| hsa01522 | Endocrine resistance | 0.003 | 0.049 | ESR1/PIK3R1/MDM2/GRB2/ADCY8/RAF1/MAPK10/ADCY4 |
| hsa04750 | Inflammatory mediator regulation of TRP channels | 0.003 | 0.049 | CALM2/PIK3R1/CALML4/ADCY8/PLCB1/PLA2G4A/MAPK10/ADCY4 |
| hsa05231 | Choline metabolism in cancer | 0.003 | 0.049 | CHPT1/RALGDS/PIK3R1/GRB2/RAF1/PLPP2/PLA2G4A/MAPK10/ |

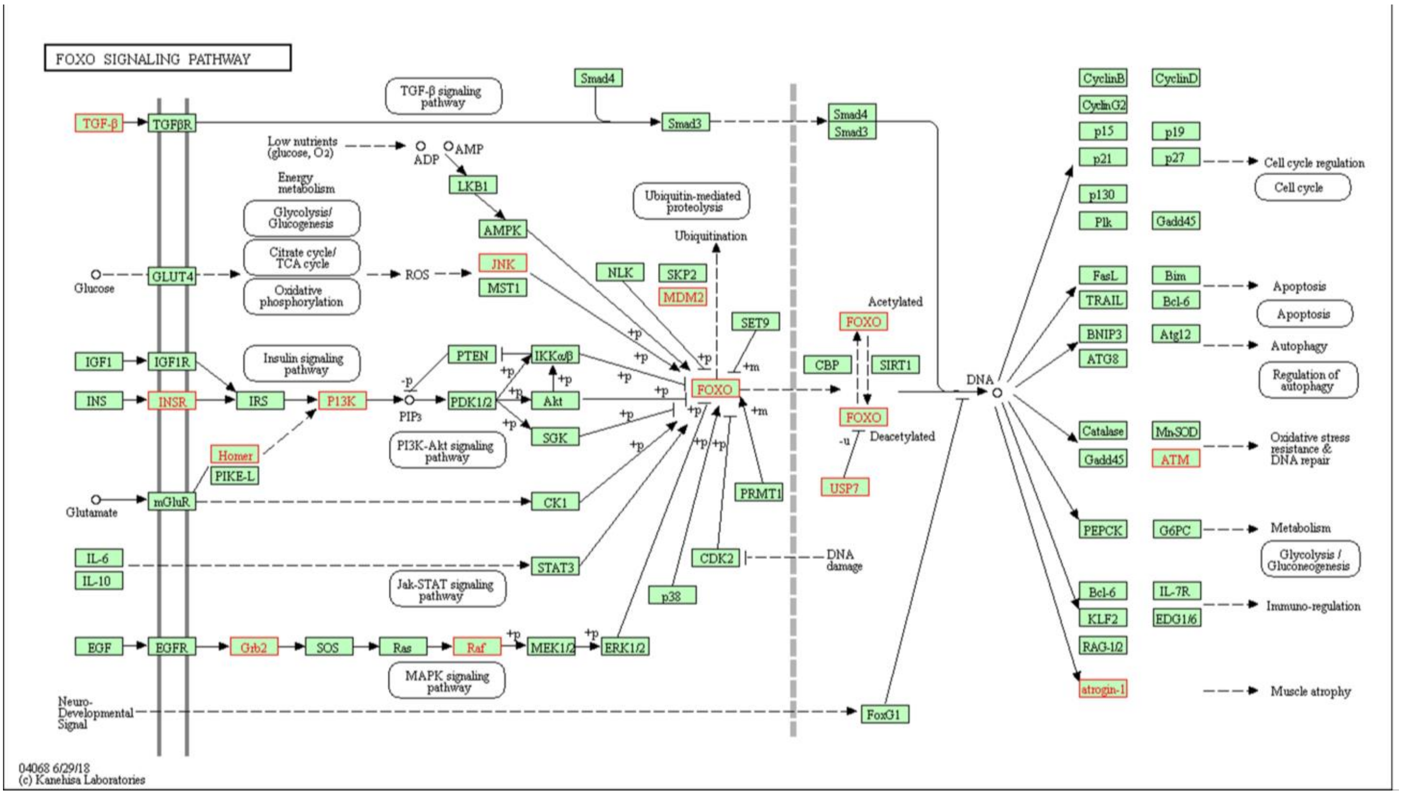


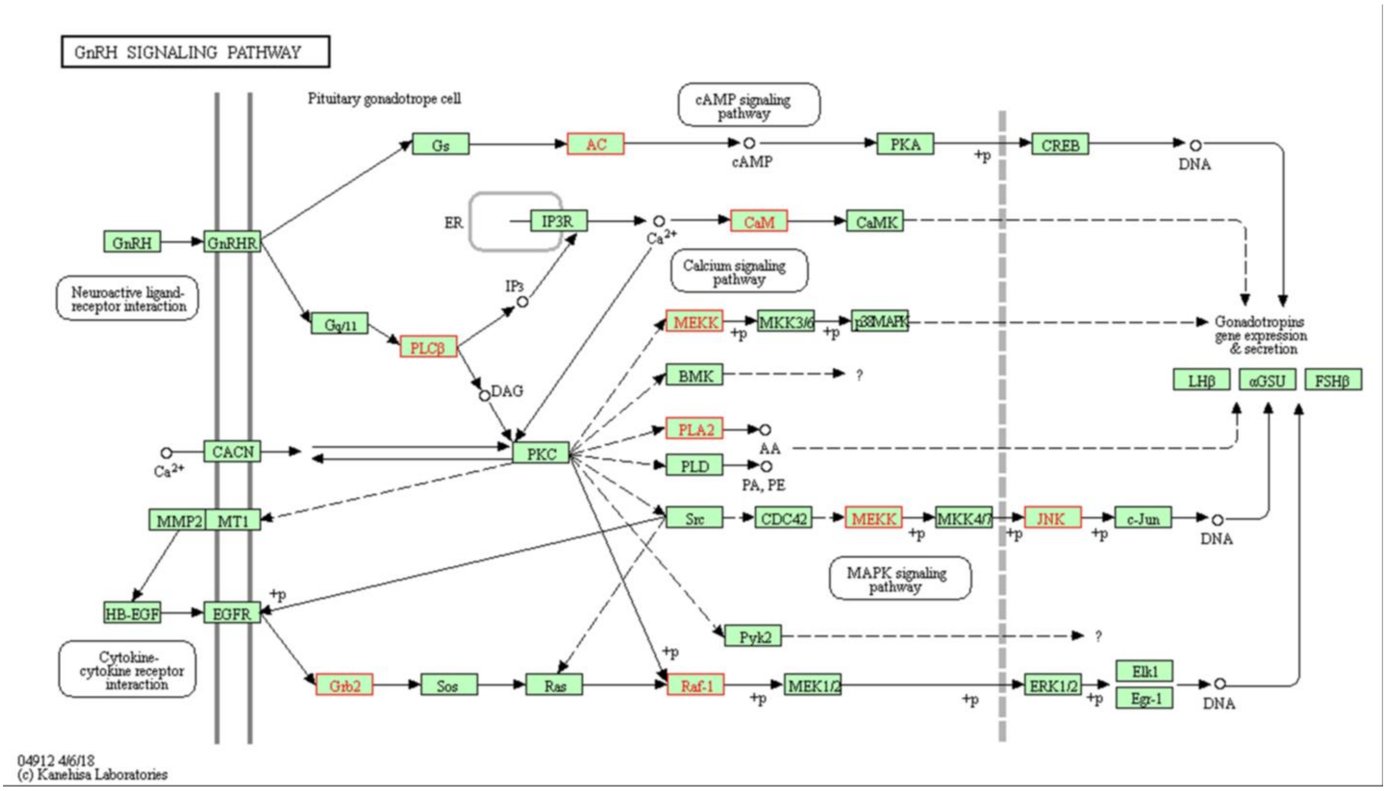


**Supplementary Figure S5.** Significant pathway 'FoxO signaling pathway' and 'GnRH signaling pathway' from KEGG database (adjusted p-value for multiple comparisons: 0.013)(printed with copyright permission of KEGG).

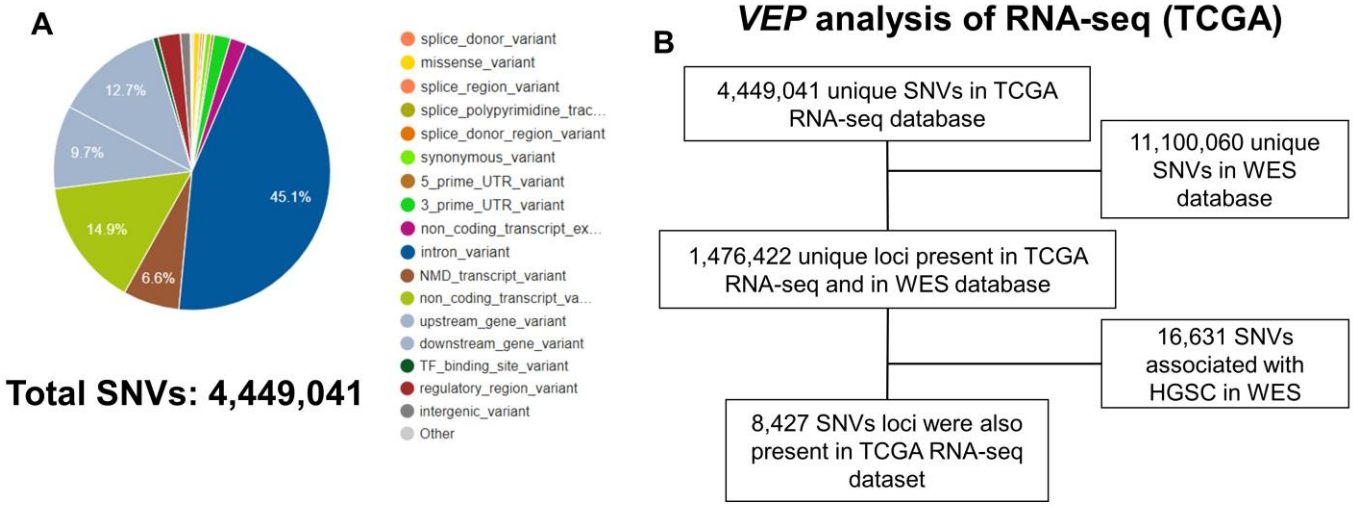


**Supplementary Figure S6.** Analysis of all SNVs from all TCGA HGSC RNA-seq dataset analyzed with VEP. A. Distribution of all predicted functional consequences of the SNVs found in a pie format. B. Of the initial almost 4.5 million SNVs found in all TCGA HGSC samples, over 1.4 million were also present in WES VEP analysis (and not in the gnomAD database, Fig. 1). Also, out of the initial 16,631 selected SNVs, associated with HGSC in the WES VEP analysis, 8,427 SNVs were also present in TCGA RNA-seq VEP analysis. Unfortunately, there are no normal samples that were sequenced in TCGA to use as controls.

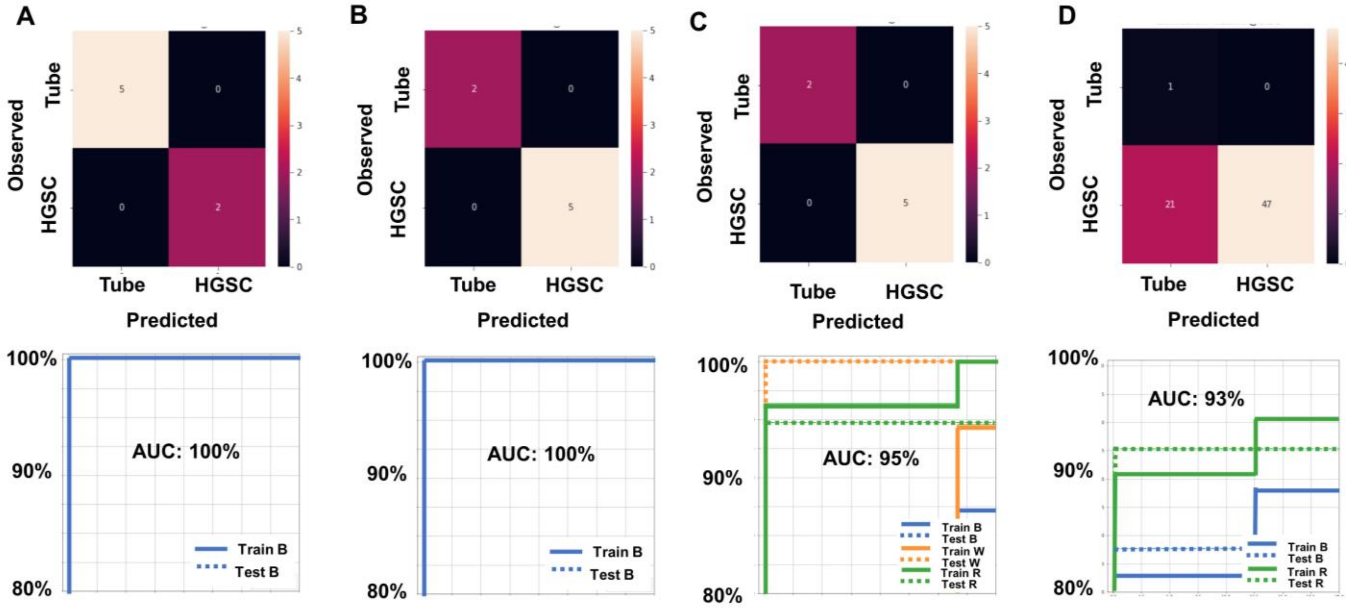


**Supplementary Figure S7.** Validation of WES DNA SNV prediction model of HGSC performed in RNA-seq samples with machine learning analytical platform.

**A.** Model with all SNV associated with HGSC (N=16,631). The superior panel shows the confusion matrix representing the observed versus the predicted values. The inferior panel is an ROC graphic: true positives in the x axis, false positives in the y axis, and AUC results. Train B: results of baseline training; Test B: results of baseline testing. **B.** Model resulting from the multivariate analysis with lasso (N=49). Superior and inferior panels are as before. **C.** Model with UI RNA-seq SNVs (N=20). Superior panel is as before. Inferior panel represents the ROC graphic including models accounting for weights of the outcome: 1) Train W: results of weighted model training; Test W: results of weighted model testing; 2) Train R: results of unbalanced (or re-sampling) model training; Test R: results of re-sampling model testing. **D.** Model with TCGA RNA-seq SNVs (N=18). Superior panel is as before. Inferior panel represents the ROC graphic including models accounting for weights of the outcome: Train R: results of unbalanced (or re-sampling) model training; Test R: results of re-sampling model testing.

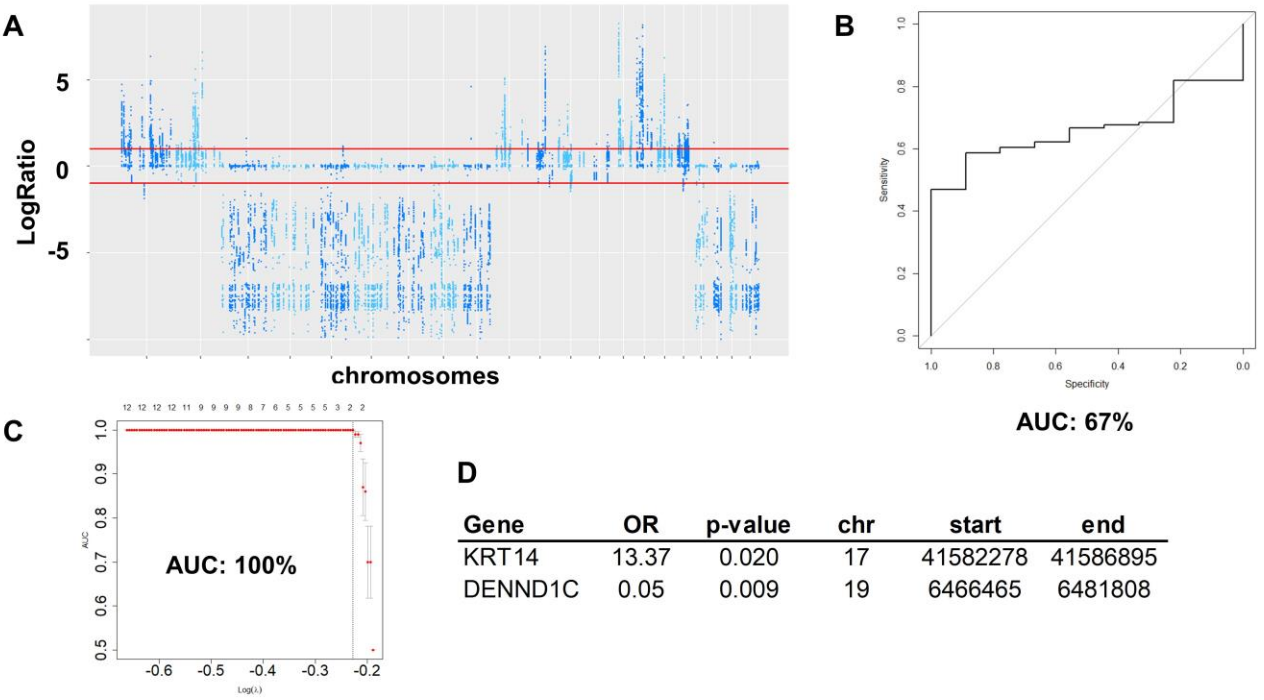


| Gene | OR | p-value | chr | start | end |
|---|---|---|---|---|---|
| KRT14 | 13.37 | 0.020 | 17 | 41582278 | 41586895 |
| DENND1C | 0.05 | 0.009 | 19 | 6466465 | 6481808 |

**Supplementary Figure S8.** Validation of WES DNA CNV analysis performed in RNA-seq samples.

**A.** Manhattan plot representations of all 558 CNV significant in the WES and assessed in the RNA-seq experiment. The red lines represent x2 copies (or more than diploid) and 0.5 copies (or less than heterozygous). There are specific gains and losses in some chromosomes: gains in 1,2,10,11,12,15,16,17, and 19; losses in 3,4,5,6,7,8,9,20,21,22, and X.

**B.** Validation of the DNA lasso prediction model in RNA-seq data (Fig. 6C), with a fair performance of an AUC of 66%: for a sensitivity of 85% the accuracy was 89% and PPV of 92%.

**C.** CNV lasso prediction regression analysis performed in RNA-seq samples, not using the lasso model from DNA analysis. ROC curve of this prediction model with 2 genes with CNV: AUC of 100%, (95% CI:100,100). These two CNV, CNTN4-AS1 and ENST00000565823 are protective for HGSC.

**D.** Logistic regression analysis including the significant 558 CNV in the WES analysis. This analysis was done independently of the WES DNA model. One gene increased the risk, KRT14, the other one decreased, DENND1C.

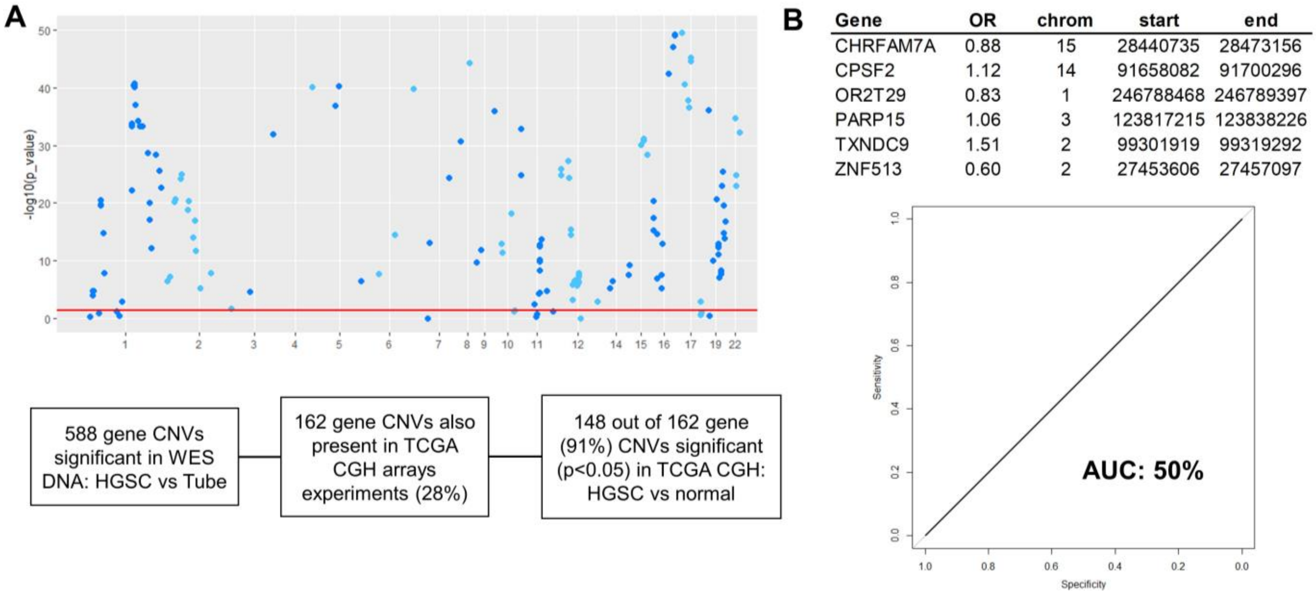


**Supplementary Figure S9.** Validation of WES DNA CNV analysis performed in TCGA RNA-seq samples.

**A.** Manhattan plot representations of 162 CNV present in TCGA CGH database, out of the 558 significant CNV in the WES DNA analysis. The red lines represent p-value <0.05. 148 were significant out of 162. **B.** Validation of the DNA lasso prediction model in TCGA RNA-seq data. The WES DNA model was re-done with the 162 genes present in the CGH array TCGA database. The table is the model in WES DNA, that had an AUC of 80% (95% CI 74%, 86%), lower than the full model with 588 genes (87% in Fig. 6). The ROC represents the validation in TCGA dataset with an AUC of 50%, for a sensitivity of 85%, accuracy of 51% and PPV of 51%.

A

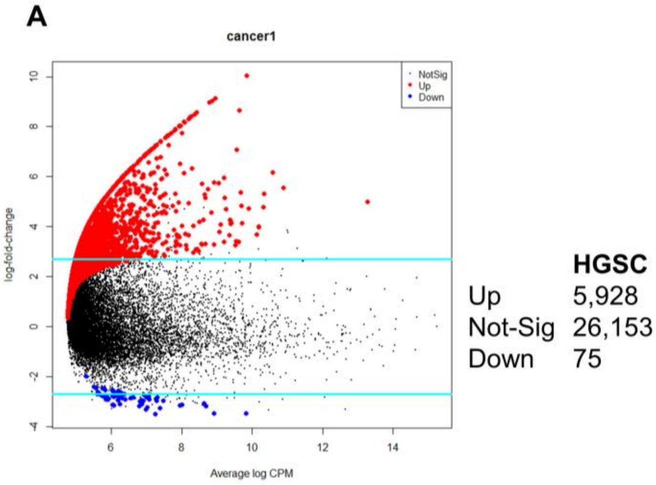


B

| Structural Variant (SV) type | Number |
|---|---|
| AS (alternative splicing) -- spliced contig alignment where both junctions are known reference exon boundaries, but spliced reads across these boundaries have not previously been observed. | 170 |
| DEL (deletion) -- aligned section of contig that does not contain part of the reference sequence. | 126 |
| EE (extended exon) -- contig alignment extends past an exon boundary. | 134 |
| FUS (fusion) -- contig involving hard-clipping (one part of the contig aligns to one part of the genome, and the other part aligns elsewhere). Fusions require at least one gene to be involved in all cryptic variants. | 480 |
| IGR (intragenic rearrangement) -- hard clipped contig where both parts align within the same gene(s). | 135 |
| INS (insertion) -- aligned section of the contig that is not in the reference. This could be a tandem duplication, but is not annotated as such yet | 208 |
| NE (novel exon) -- an aligned block that does not cross any existing exons. May be intronic or intergenic. | 2164 |
| NEJ (novel exon junction) -- spliced contig where neither junction boundary matches the reference. | 1046 |
| PNJ (partial novel junction) -- spliced contig where one junction matches a known boundary (the other side is unknown). | 88 |
| RI (retained intron) -- a contig block that spans a whole intron. | 436 |
| UN (unknown) -- by default, all soft-clipped contigs are classified as unknown at the moment. These variants need to be better annotated. | 4462 |

**Supplementary Figure S10.** Univariate analysis of all SV for cases vs controls (package *edgeR*). **A.** Out of 32,156 SVs, 6,003 were below the cut-off in multiple univariate analyses (at p<0.001). 75 of them were decreased in HGSC and 5,928 were increased. **B.** The table details the type and number of SV that were significant.

**A**

| SV | chr | start | size | strand | SV type | genes | OR | p-value | 95% CI |
|---|---|---|---|---|---|---|---|---|---|
| chr3_147402104_RI | chr3 | 147402104 | 624 | + | RI | ZIC4 | 1.53 | 0.012* | 1.16, 2.35 |
| chr4_157677227_NE | chr4 | 157677227 | 107 | + | NE | LOC105377509 | 1.34 | 0.050* | 1.01, 1.90 |

**B**

| Loci | chr | start | size | strand | SV type | genes | OR | p-value | 95% CI |
|---|---|---|---|---|---|---|---|---|---|
| chr8_69118424_NE | chr8 | 69118424 | 86 | - | NE | LINC01592 | 1.29 | 0.148 | 0.97, 3.75 |
| chr4_157677227_NE | chr4 | 157677227 | 107 | + | NE | LOC105377509 | 1.34 | 0.050* | 1.01, 1.90 |

**C**

| SV | chr | start | size | strand | SV type | genes | OR | p-value | 95% CI |
|---|---|---|---|---|---|---|---|---|---|
| chr20_63241753_RI | chr20 | 63241753 | 709 | + | RI | NKAIN4 | 1.38 | 0.151 | 0.94, 2.39 |
| chr3_147402104_RI | chr3 | 147402104 | 624 | + | RI | ZIC4 | 1.54 | 0.011* | 1.17, 2.36 |

**D**

| SV | chr | start | size | strand | SV type | genes | OR | p-value | 95% CI |
|---|---|---|---|---|---|---|---|---|---|
| chr11_2149345_PNJ | chr11 | 2149345 | 127 | + | PNJ | IGF2\|IGF2-AS\|INS-IGF2 | 1.96 | 0.047* | 1.20, 5.39 |
| chr12_21334709_PNJ | chr12 | 21334709 | 365 | + | PNJ | SLCO1A2 | 2.43 | 0.302 | 0.78, 85.11 |
| chr8_139601601_PNJ | chr8 | 139601601 | 457 | + | PNJ | KCNK9 | 1.67 | 0.106 | 1.03, 4.11 |

**E**

| SV | chr | start | size | strand | SV type | genes | OR | p-value | 95% CI |
|---|---|---|---|---|---|---|---|---|---|
| chr12_57216843_UN | chr12 | 57216843 | 40 | - | UN | NXPH4 | 1.38 | 0.212 | 0.95, 3.13 |
| chr14_105863814_UN | chr14 | 105863814 | 36 | + | UN | ADAM6\|LINC00221 | 1.52 | 0.045* | 1.08, 2.62 |

**Supplementary Figure S11.** Multivariate analysis of all SV for cases vs controls. **A.** Multivariate analysis with all 6,003 selected SV in multiple univariate analyses. **B.** Multivariate analysis with only novel exon (NE) selected SV in univariate analyses. **C.** Multivariate analysis with only retained intron (RI) selected SV in univariate analyses. **D.** Multivariate analysis with only partial novel junction (PNJ) selected SV in univariate analyses. E. Multivariate analysis with only unknown (UN) selected SV in univariate analyses.

SV: structural variant. NE: novel exon -- an aligned block that does not cross any existing exons. May be intronic or intergenic. RI: retained intron -- a contig block that spans a whole intron. PNJ: partial novel junction -- spliced contig where one junction matches a known boundary (the other side is unknown). UN: unknown -- by default, all soft-clipped contigs are classified as unknown at the moment. * Significant at a p-value < 0.05.

**A**

| SV | OR |
|---|---|
| chr10_101729717_NE | 1.95 |
| chr11_2149345_PNJ | 1.52 |
| chr11_89498187_AS | 1.54 |
| chr13_55179004_NE | 1.11 |
| chr14_105743009_NE | 1.17 |
| chr14_105863198_NE | 1.08 |
| chr14_105863814_UN | 1.14 |
| chr14_105986593_FUS | 1.11 |
| chr19_8876368_IGR | 1.31 |
| chr20_33053965_NEJ | 1.63 |
| chr21_8259383_NE | 1.52 |
| chr3_147402104_RI | 1.35 |
| chr5_102458491_AS | 1.29 |
| chr5_102608496_INS | 1.22 |
| chr5_148089622_EE | 1.16 |
| chr5_85663902_NEJ | 1.77 |
| chr6_170156045_AS | 1.32 |

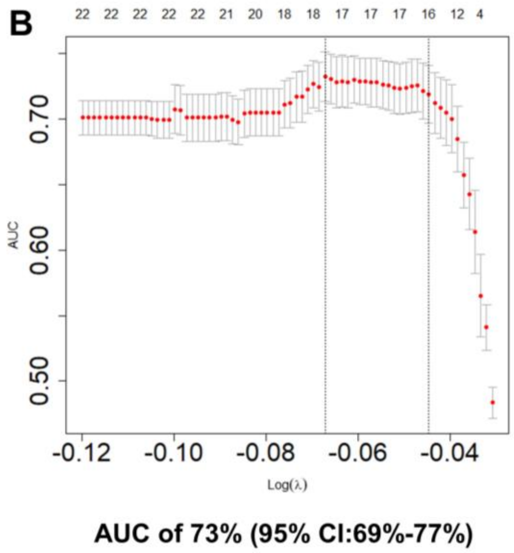


**Supplementary Figure S12.** Multivariate Lasso prediction model of HGSC with SVs. 22 SV were initially selected in ANOVA univariate analyses with cross-validation out of the total 32,156 SVs. **A.** The lasso multivariate prediction analysis identified 17 SV that predicted HGSC with an AUC of 73%. **B.** Prediction model with performance by AUC of 0.73 (95% CI: 69%-77%).

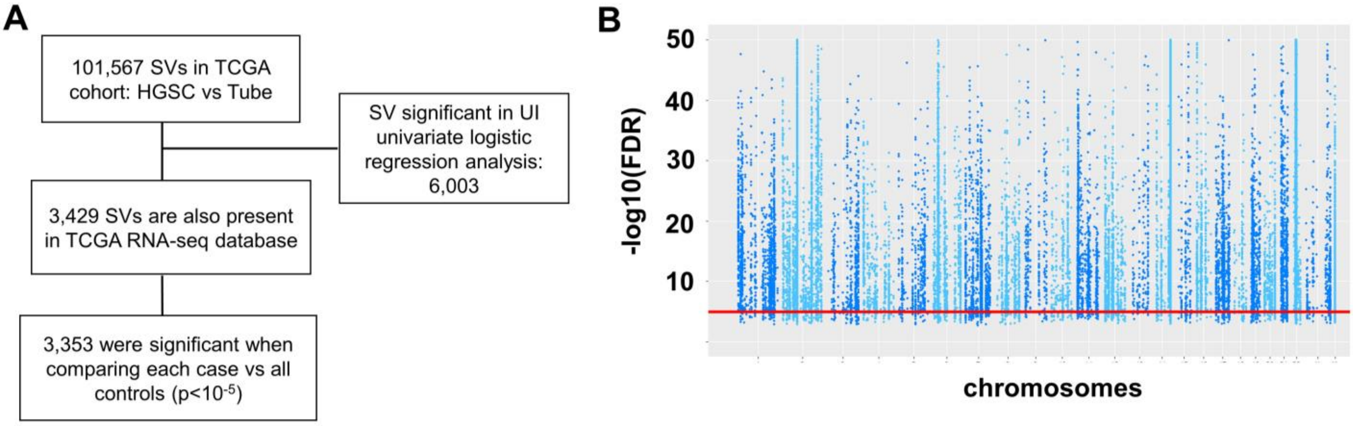


**C**

| SV | chr | start | size | strand | SV type | genes | OR | p-value | 95% CI |
|---|---|---|---|---|---|---|---|---|---|
| chr11_2149345_PNJ | chr11 | 2149345 | 127 | + | PNJ | IGF2\|IGF2-AS\|INS-IGF2 | 3.08 | 0.056 | 1.58, 34.63 |
| chr14_105863814_UN | chr14 | 105863814 | 36 | + | UN | ADAM6\|LINC00221 | 1.14 | 0.068 | 1.002, 1.35 |

**Supplementary Figure S13.** Validation of structural variation (SV) between HGSC samples and tubal controls in TCGA dataset. Analysis performed with MINTIE. Controls were taken from same tubal RNA-seq experiments used for the UI dataset. **A.** All SV for each individual case (371), each one compared to controls (12). 3,429 SVs out of 6,003 selected in the UI analysis, were also present in TCGA dataset. 3,353 of those were significant when compared with controls in the MINTIE analysis, with a FDR <0.05 and log2 fold change >2. **B**. Selected SV are represented in a Manhattan plot. **C.** Multivariate analysis with all SV significant in the UI multivariate analysis: 2 SV were independently significant.

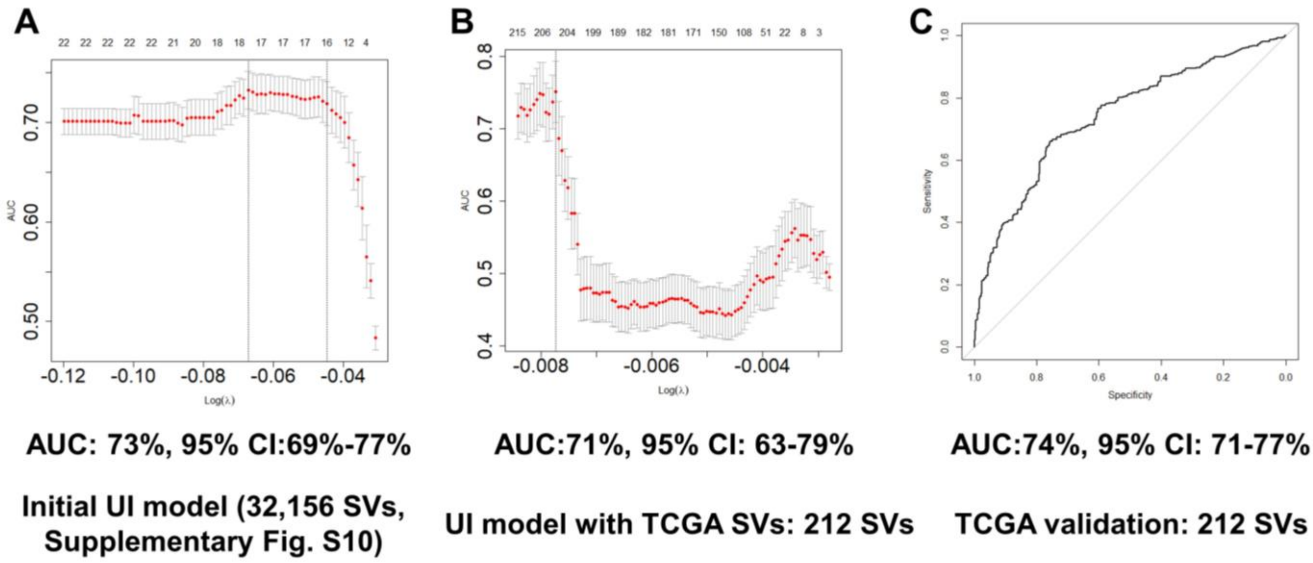


**Supplementary Figure S14.** Validation of multivariate lasso prediction analysis of structural variation (SV). **A.** Original lasso prediction model of HGSC with final 17 SV included in the model. **B.** UI lasso prediction model of the 3,429 SVs (out of 6,003) that were selected and present in TCGA database. The prediction model was comprised of 212 SVs for an AUC of 71%. **C.** Validation of the UI multivariate analysis in TCGA dataset, with a good performance of an AUC of 74%, accuracy of 63%, sensitivity of 85%, and PPV of 73%.
